# Supplementary material for: Manuka Honey Inhibits Human Breast Cancer Progression in Preclinical Models
Source: Nutrients. 2024 Jul 22;16(14):2369. doi: 10.3390/nu16142369 (PMC11279598; doi:10.3390/nu16142369)
Supplement: Supplementary file 1 [file nutrients-16-02369-s001.zip › nutrients-3076621-supplementary.pdf]

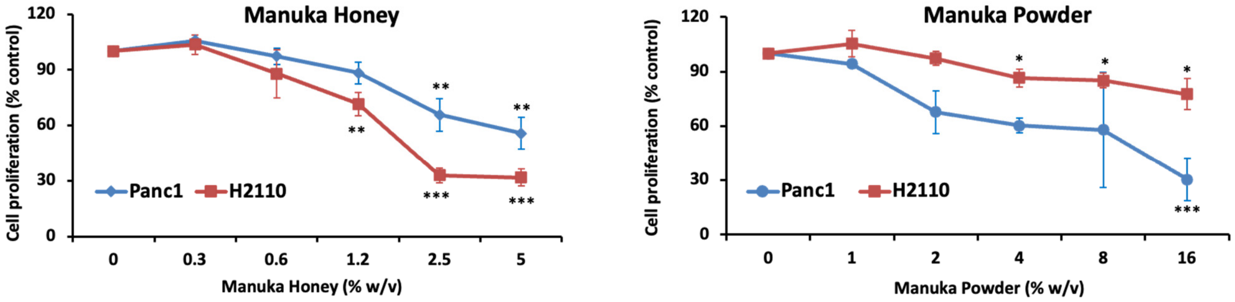

Figure S1. Manuka honey reduces the proliferation of Pancreatic and non-small cell lung cancer cells. Panc1 pancreatic cancer cell and H2110 non-small cell lung cancer cells were incubated in the presence of increasing concentrations of either: (**A**) Manuka Honey at 0.0 to 5.0 % (w/v) or (**B**) Manuka powder at 0.0 – 16%. After 72 hours, cell counts were done using an MTS assay and by manual cell counts. The figures show tumor cell proliferation expressed as the mean percentage of vehicle-treated controls with SEM. Experiments were performed at least three times in independent experiments. *P< 0.05, **P< 0.01, ***P< 0.001.

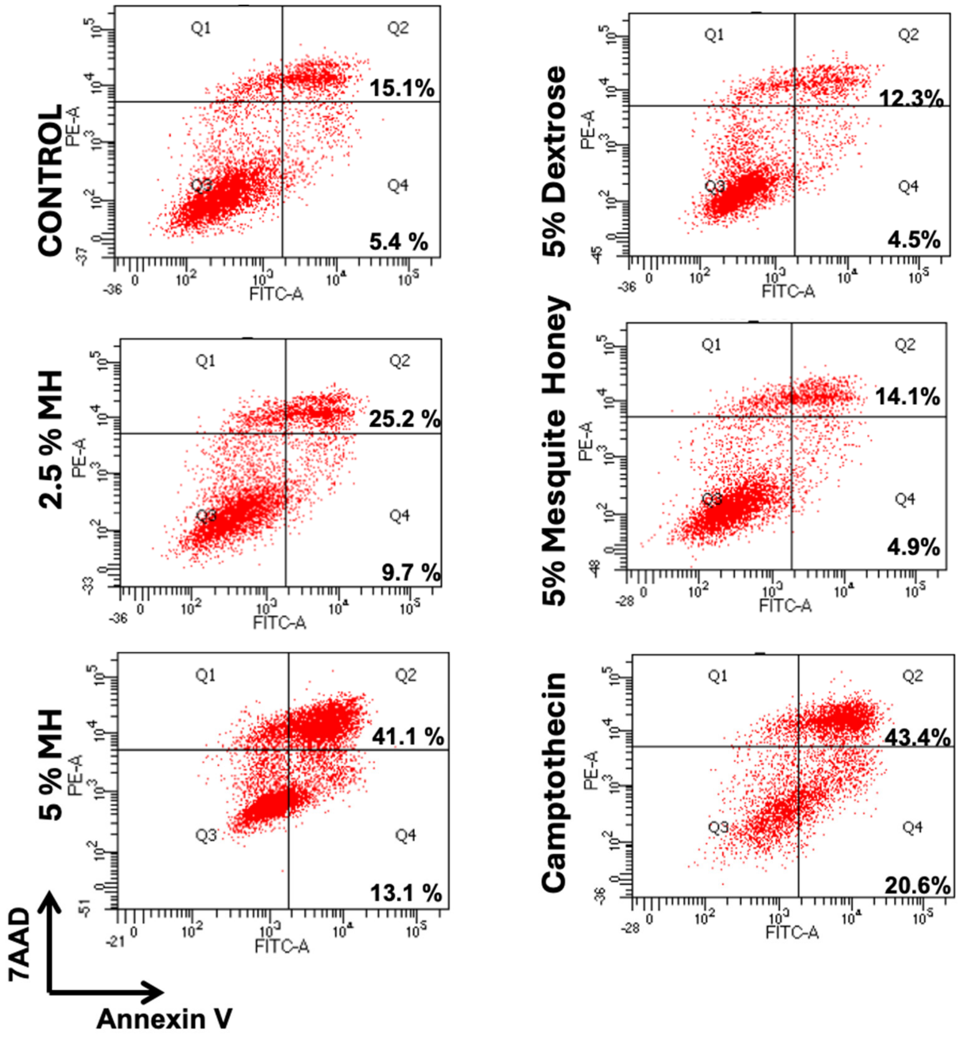

Figure S2. Induction of apoptosis of breast cancer cells by Manuka honey. A) MDAMB231 triple negative breast cancer cells were treated with vehicle control, 2.5% (w/v) (2.5 MH) or 5% (w/v) (5.0 MH) manuka honey, camptothecin 1 µM (Camptothecin), 5% (w/v) dextrose or 5% (w/v) mesquite honey. After 48 hours, cells were harvested and stained with Annexin V and 7-AAD to assess early and late apoptosis. Treatments with Manuka honey, particularly at 5.0 % (w/v) elicited increments in early and late apoptotic cells as compared to controls (*P<0.05). Camptothecin, a positive control drug, elicited a similar increase in late apoptotic cells after 48 hours, while treatment of MDAMB231 cells with dextrose or mesquite honey did not exhibit comparable increments in the numbers of apoptotic cells. Figure shows one representative experiment.
